# Tentacle Transcriptome and Venom Proteome of the Pacific Sea Nettle, *Chrysaora fuscescens* (Cnidaria: Scyphozoa)

**DOI:** 10.3390/toxins8040102

**Published:** 2016-04-05

**Authors:** Dalia Ponce, Diane L. Brinkman, Jeremy Potriquet, Jason Mulvenna

**Affiliations:** 1 Australian Venom Research Unit, Department of Pharmacology and Therapeutics, University of Melbourne, Melbourne, Victoria 3010, Australia; d.poncegarcia@student.unimelb.edu.au; 2 Australian Institute of Marine Science, Townsville, Queensland 4810, Australia; 3 Queensland Institute of Medical Research (QIMR) Berghofer Medical Research Institute, Infectious Diseases Program, Brisbane, Queensland 4006, Australia; jeremy.potriquet@qimrberghofer.edu.au; 4 School of Biomedical Sciences, The University of Queensland, Brisbane, Queensland 4072, Australia

**Keywords:** Jellyfish, *Chrysaora*, venom, transcriptome, proteome, toxin, nematocyst

## Abstract

Jellyfish venoms are rich sources of toxins designed to capture prey or deter predators, but they can also elicit harmful effects in humans. In this study, an integrated transcriptomic and proteomic approach was used to identify putative toxins and their potential role in the venom of the scyphozoan jellyfish *Chrysaora fuscescens*. A *de novo* tentacle transcriptome, containing more than 23,000 contigs, was constructed and used in proteomic analysis of *C. fuscescens* venom to identify potential toxins. From a total of 163 proteins identified in the venom proteome, 27 were classified as putative toxins and grouped into six protein families: proteinases, venom allergens, C-type lectins, pore-forming toxins, glycoside hydrolases and enzyme inhibitors. Other putative toxins identified in the transcriptome, but not the proteome, included additional proteinases as well as lipases and deoxyribonucleases. Sequence analysis also revealed the presence of ShKT domains in two putative venom proteins from the proteome and an additional 15 from the transcriptome, suggesting potential ion channel blockade or modulatory activities. Comparison of these potential toxins to those from other cnidarians provided insight into their possible roles in *C. fuscescens* venom and an overview of the diversity of potential toxin families in cnidarian venoms.

## 1. Introduction

Phylum Cnidaria is the largest and most diverse group of venomous marine invertebrates and includes five Classes: Anthozoa (sea anemones, corals), Cubozoa (box jellyfish), Hydrozoa (hydras, hydroids), Scyphozoa (true jellyfish) and Staurozoa (stalked jellyfish). The phylum is divided into two subphyla, Anthozoa and Medusozoa (comprising the four remaining Classes), based on differences in mitochondrial genomes and life cycles [1,2].

The venomous nature of cnidarians is defined by the nematocyst, a complex intracellular structure that injects a venomous cocktail into prey or predators that come in contact with the tentacles or other body parts of cnidarians. Nematocyst venoms are mixtures of peptides, proteins and other components that can cause cytotoxicity, ion channels blockade, membrane pore formation, *in vivo* cardiovascular collapse and lethal effects in experimental animals (reviewed in [3,4,5,6]).

**Figure 1:**
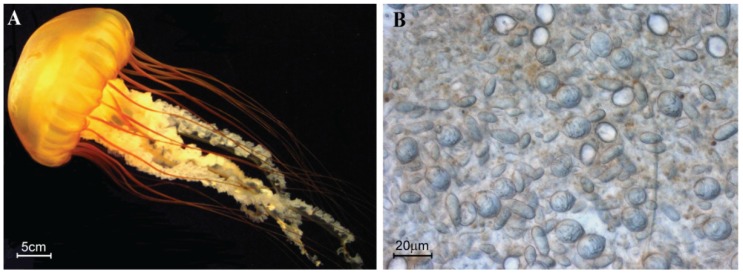
*C. fuscescens* jellyfish and tentacle tissue. (**A**) Representative adult specimen used for biological sampling. Average bell diameter of mature medusae is 25 cm and extended tentacles are approximately 1 m long. Photograph © Dalia Ponce; (**B**) Micrograph of the tentacle tissue used for RNA extractions and nematocyst isolation. Photograph © Dr. Diane Brinkman.

Scyphozoan jellyfish have a worldwide distribution and are considered to be mild to severe stingers. In particular, jellyfish from the genus *Chrysaora* (sea nettles) inflict stings that can cause harmful reactions in humans including burning sensation, blisters, skin redness, headaches, cramps and lachrymation [7,8]. *Chrysaora fuscescens* (Figure 1) is common on the western seaboard of Canada, United States of America and Mexico and despite possessing a painful sting [9], no study has been devoted to characterization of its venom. Early studies have examined some other *Chrysaora* venoms [10,11], particularly from *Chrysaora quinquecirrha*, which can cause cardiotoxicity, dermonecrosis, myotoxicity, haemolysis, neurotoxicity, hepatotoxicity and lethality in experimental animals [12,13,14,15,16,17,18,19]. Although these reports describe the clinical and experimental effects of some *Chrysaora* venoms, the molecular mechanisms underlying these toxic effects are poorly understood, partly because the composition of sea nettle venoms has not been fully elucidated and individual toxin components have not been characterized.

Studies estimate that more than 25,000 potential toxins from cnidarian venoms are yet to be explored as potential therapeutics, novel templates for drug design or diagnostic tools [20,21]. Identification of individual toxins has been difficult mainly due to the high amount of venom sample required for isolation and characterization using classical biochemistry techniques (e.g., bioassay-guided fractionation) [22], and to the intrinsic instability of cnidarian venom proteins [6]. Significant progress in the profiling of cnidarian venoms has recently been achieved using combined proteomic and transcriptomic analyses, which have enabled the identification of putative toxins and their potential roles in venoms [23,24,25,26]. However, due to the limited number of available reference genomes for cnidarians (*Nematostella vectensis* [27], *Hydra vulgaris* (synonym: *Hydra magnipapillata*) [28] and *Acropora digitifera* [29]), most of these studies have employed a strategy of *de novo* transcriptome assembly from short reads (despite its associated challenges [26,30]) to identify putative toxin families and nematocyst-related proteins [23,24,26]. This approach can be combined with proteomic characterisation of venom proteins where the use of species-specific transcriptomes improves protein identifications compared to public database searches [31]. Accordingly, in this work we employed a similar workflow in which transcriptome sequence data generated by Next Generation Sequencing (NGS) was combined with proteomic interrogation of *C. fuscescens* venom to identify potential toxins and other nematocyst components. These results add to the growing catalogue of jellyfish venom proteins and will assist in the design of targeted experiments to further isolate and characterize specific proteins. Finally, the identification of putative venom proteins can also give clues on the evolutionary diversification of toxins and different strategies for prey capture or predator deterrence, which will lead to a better understanding of the toxinology of cnidarians.

## 2. Results and Discussion

### 2.1. Construction of a Protein Database from the C. fuscescens Tentacle Transcriptome

NGS and *de novo* assembly were used to construct a *C. fuscescens* tentacle transcriptome to identify putative toxins at the transcript level and to provide protein sequences for proteomic interrogation of *C. fuscescens* venom. More than 26 million Illumina paired-end raw reads were used in Trinity [32] to assemble a transcriptome composed of 30,317 contigs with an average length of 628 bases (Table 1, Figure S1). ESTScan analysis, using a cnidarian matrix previously generated in-house from cnidarian sequences from the EMBL and GenBank databases [26], identified coding DNA sequences (CDS) in 78% of the assembled contigs.

**Figure 2:**
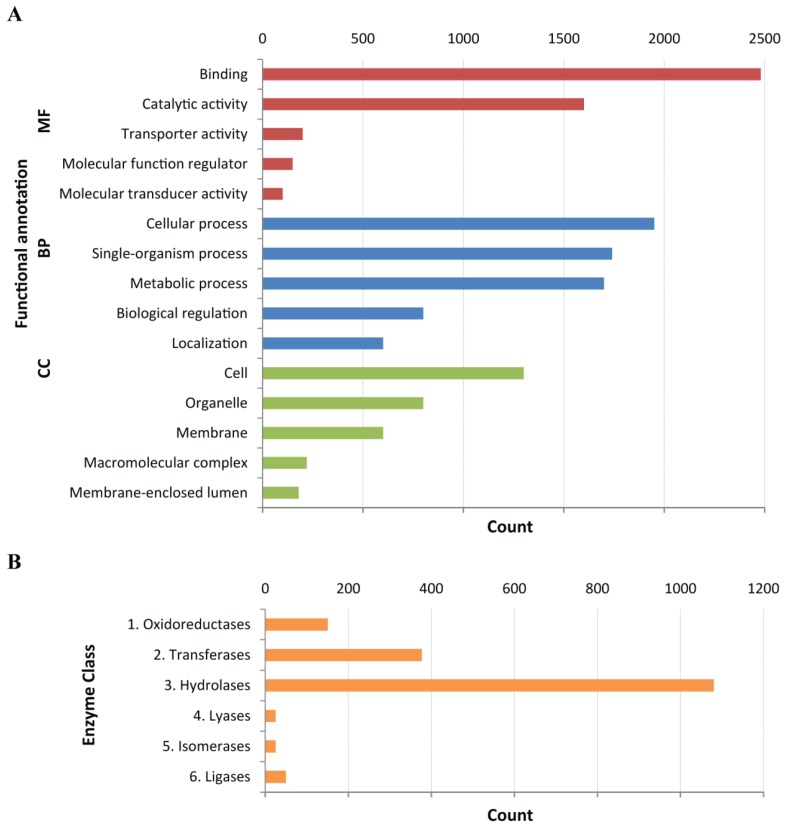
Functional annotation of transcripts with predicted coding regions. (**A**) Top 5 GO term hits in the categories of molecular function (MF), biological process (BP) and cellular component (CC); (**B**) Classification of enzymes according to Enzyme Commission (EC) number.

To provide an annotated protein database for proteomics analysis, these coding regions were compared to sequences available in a set of public databases using BLASTx (see Section 4.4) and then functionally annotated using a combination of InterProScan [33] and Blast2GO [34]. Gene Ontology (GO) terms were assigned to 11,586 CDS (49%) and those under the “Molecular Function” category were the most highly represented (35%) (Figure 2A). Approximately 1,700 CDS were identified as coding for enzymes and classified according to their Enzyme Commission (EC) numbers using Blast2GO; the majority of which (~1100) were hydrolases (Figure 2B). Transcriptome analysis and protein coding sequence detection resulted in a protein database composed of 23,534 protein sequences for use in proteomics experiments. A similar number of transcripts with predicted coding regions from *de novo* transcriptomes have been used for toxin identification in the cubozoan jellyfish *Chironex fleckeri* [26] and the scyphozoans *Stomolophus meleagris* [23] and *Cyanea capillata* [35].

### 2.2. Proteomic Analysis of C. fuscescens Venom

**Figure 3:**
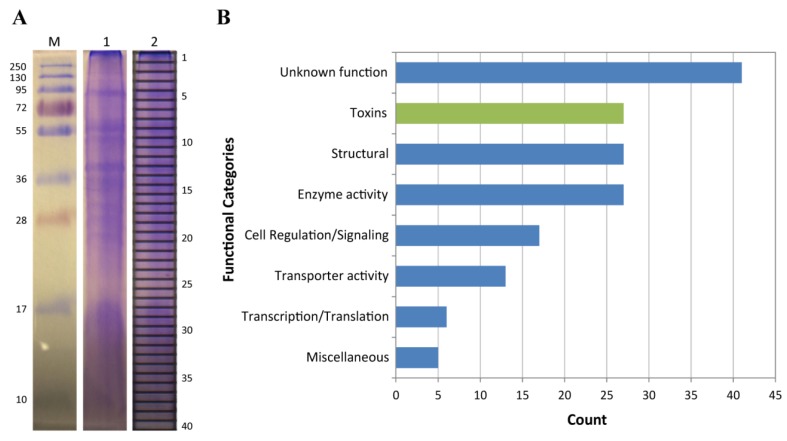
Venom proteome of *C. fuscescens*. (**A**) SDS-PAGE analysis of crude venom (lanes 1 and 2). The 40 gel bands used for in-gel tryptic digestion and tandem mass spectrometry are indicated in lane 2. Molecular masses of the protein marker (M) are shown alongside in kDa; (**B**) Functional annotation of proteins identified in proteomics experiments.

To identify toxin proteins in *C. fuscescens* venom, crude venom was fractionated using SDS-PAGE (Figure 3A) and peptides from in-gel tryptic digests were analyzed using tandem mass spectrometry (MS/MS). Spectra from tandem MS experiments were searched against the *C. fuscescens* protein database described above. A total of 163 proteins were identified and these were grouped according to their functional annotation (Table S1, Figure 3B). From these annotated proteins, the most highly represented were toxin-like proteins, enzymes and structural proteins. Potential toxins were identified on the basis of manual annotations using BLAST to identify proteins with homology to described toxins in the UniProt animal toxin and venom database, the results of GO annotations, the lack of higher-scoring BLAST hits to non-toxin proteins in UniProt and described toxic activity in other jellyfish species. The final set of potential toxins included fourteen proteases, six cysteine-rich secretory proteins (CRISPs), three C-type lectins, two pore-forming toxins, one glycoside hydrolase and a protease inhibitor (Table 2). This pattern of toxin family distribution is similar to the venom proteomes of other medusozoans, including *Olindias sambaquiensis*, *H. vulgaris* and *C. fleckeri*, in which the most abundant venom proteins identified were proteases [20,36], pore-forming toxins [37] followed by CRISPs, protease inhibitors and lectins [26].

Twenty-seven enzymes were identified in the *C. fuscescens* venom proteome which were mainly proteinases, oxido-reductases and glycosidases (Table S1). Twenty-seven structural proteins were identified, including nematocyst-specific proteins such as NOWA and nematogalectins that have been also been reported in the venom proteomes of *H. vulgaris* [36] and *C. fleckeri* [37]. The remaining components identified in the proteome included proteins associated with cell regulation (17), transporter activities (13), transcription/translation processes (6) and miscellaneous functions (5). Forty-one proteins (25%) had unknown functions, which highlights the significant proportion of cnidarian proteins that are yet to be characterized. Further searches conducted against cnidarian protein sequences from GenBank identified two additional proteins, a 40S ribosomal protein and a heat shock protein, neither of which was considered as a potential toxin.

#### 2.2.1. Proteases

Proteases are important protein toxins in many venomous animals [38,39]. In the *C. fuscescens* proteome, fourteen proteases were identified representing three protease sub-types: eleven metalloproteases, two aspartyl proteases and a serine protease (Table 2). Of the metalloproteases, five were homologous to endothelin-converting enzyme (ECE) 1-like and 2-like proteins. These proteases have been identified as venom components in both *C. fleckeri* [26,37,40] and *H. vulgaris* [24,36] as well as other venomous organisms, including wasps [41] and cone snails [42]. ECEs are thought to play a supporting role in envenomation, such as acting in toxin maturation in wasps [41] or augmenting local venom concentration during cone snail stings [42]. ECEs found in medusozoan venoms could have similar functions during stings, although more experimental evidence is required. Other proteases were identified in the *C. fuscescens* venom proteome that have not been identified in the venom proteomes of other jellyfish species, including one cathepsin D-like protease, two aspartic peptidase-like proteases and a predicted PC3-like endoprotease variant B isoform X1 (Table 2). However, a cathepsin B-like protease has been identified in a cDNA library of *C. capillata* [43]. Accordingly, the repertoire of venom proteases in medusozoans is likely to be more diverse than previously understood.

#### 2.2.2. Pore-Forming Toxins

**Figure 4:**
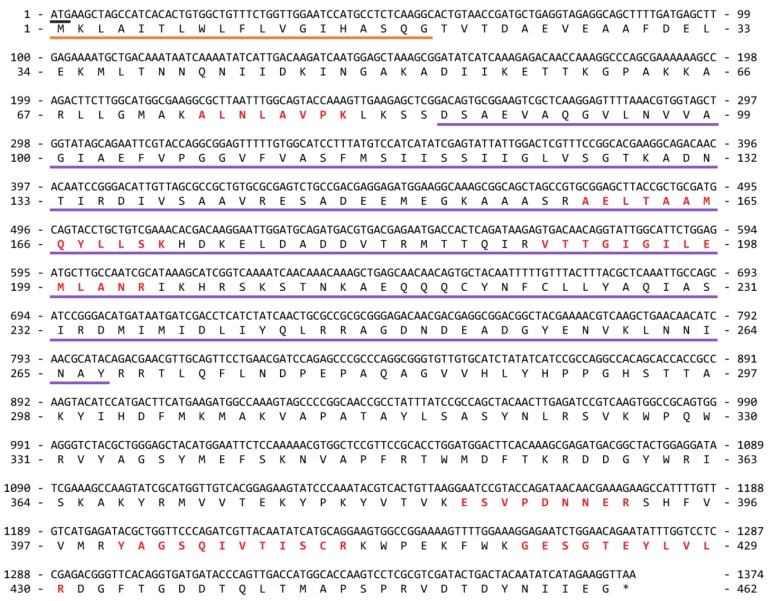
Nucleotide and deduced amino acid sequence of CfusTX-1 (GenBank accession number KU529195). The start codon (ATG) is underlined in black and the stop codon is indicated with an asterisk. A 19-residue signal peptide is underlined in orange and a predicted δ-endotoxin, *N*-terminal-like domain is underlined in purple. Unique peptide matches from mass spectrometry experiments are indicated in red.

**Figure 5:**
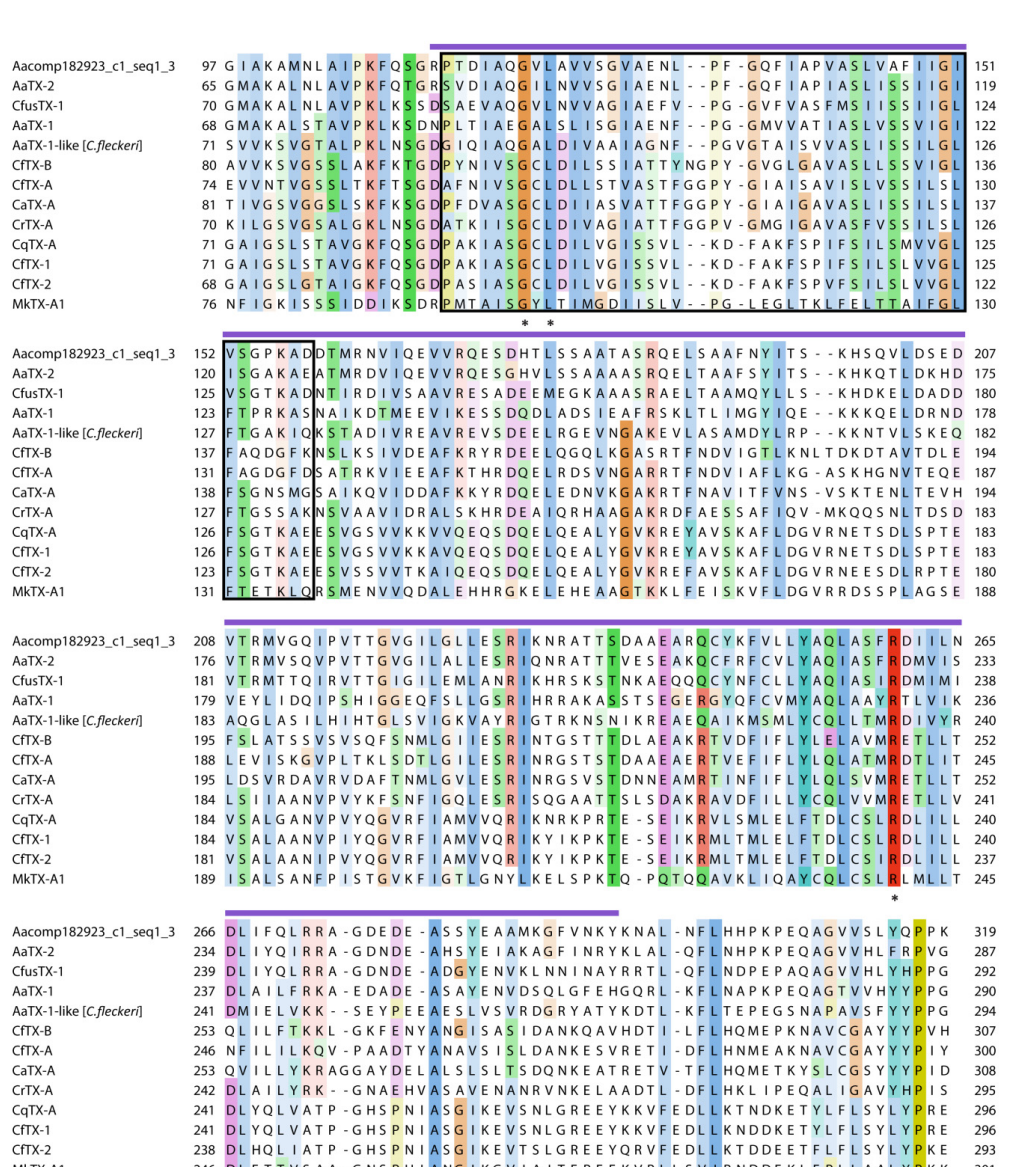
Partial multiple protein sequence alignment of CfusTX-1 and related jellyfish toxins highlighting the regions of highest sequence similarity. Sequences were aligned using MUSCLE and visualized using Jalview. Amino acid residue shading is based on the Clustal protein colour scheme, with color intensity increasing as residue conservation increases from 25% to 100%. Identical residues are indicated with an asterisk. Dashes represent gaps introduced for better alignment. A predicted transmembrane spanning region (TSR1) that is common among the jellyfish toxins is indicated with a black outline. A purple line above the alignment corresponds to a predicted δ-endotoxin, *N*-terminal-like domain. References for Aacomp182923_c1_seq1_3 [24] and AaTX-1-like [26].

Pore-forming toxins (PFTs) are common constituents in cnidarian venoms (reviewed in [3,4,5,6]). In *C. fuscescens*, two putative PFTs were identified in the venom proteome, one with sequence similarity to a growing family of jellyfish cytolysins (comp13207_c0_seq1), and the other, a novel protein (comp12925_c0_seq1). The first of these potential toxins was identified in gel bands within the 36–55 kDa molecular range and was named CfusTX-1. The full-length transcript encoding CfusTX-1 and the deduced amino acid sequence are presented in Figure 4. Sequence analysis of this protein using SignalP 4.1 [44] and InterProScan predicted the presence of a 19-residue signal peptide, indicative of a classical secretion pathway, and an *N*-terminal domain with structural homology to the *N*-terminal domain of δ-endotoxins produced by *Bacillus* bacteria (amino acid range 86–267). BLAST analysis revealed that CfusTX-1 shares high sequence similarity to predicted scyphozoan toxins, TX1 and TX2 from *Aurelia aurita*, and several related cubozoan toxins (Table 3). TMpred analysis also predicted a conserved transmembrane spanning region (TSR1) within the *N*-terminal region of CfusTX-1 (amino acids 104–125), consistent with previous reports for other related jellyfish toxins [6,35,45]. A multiple sequence alignment of CfusTX-1 with twelve related toxins from Scyphozoa and Cubozoa revealed that highest sequence similarity between homologues occurs within a 120-residue *N*-terminal region corresponding to *C. fuscescens* amino acid range 70–290, which includes both TSR1 and the δ-endotoxin *N*-terminal-like domain (Figure 5).

Members of this jellyfish toxin family were originally identified as major constituents in box jellyfish venoms [45,46,47,48,49,50,51], but in recent years, related proteins have also been identified in the venom proteomes of scyphozoans [24,35,52,53], hydrozoans [24,36] and an anthozoan [54]. Experimental studies using cubozoan venoms have demonstrated that these toxins can cause *in vitro* pore-formation, haemolysis and cytotoxicity [45,47,48,49,50,51], and *in vivo* dermonecrosis, inflammation, pain, cardiovascular collapse and death in experimental animals [45,48,49,50,51], thus implicating them as biologically important toxins in jellyfish envenomations. In the most venomous of jellyfish, *C. fleckeri*, this protein family appears to have undergone an expansion, with 15 CfTX isoforms identified in its venom proteome [26]. While this toxin family appears to be restricted to cnidarians, with no significant sequence similarity to any other protein family, several members, including the example identified here in *C. fuscescens*, are predicted to contain *N*-terminal domains with structural similarity to the *N*-terminal domains of pore-forming δ-endotoxins (Cry toxins) produced by strains of the bacterium *Bacillus thuringiensis* [6,45]. In Cry toxins, the *N*-terminal domain is involved in cell membrane insertion and pore formation [55]. Hence, the presence of a similar domain in several of the cnidarian cytolysins suggests a similar mode of pore-forming action for these proteins.

The second putative PFT identified in the *C. fuscescens* venom proteome (comp12925_c0_seq1) also contained a δ-endotoxin *N*-terminal-like domain. However, in this case, the 360 amino acid protein lacked a signal peptide and its highest BLAST match was an uncharacterized, predicted protein from *H. vulgaris* (24% identity, *E-*value = 2 × 10^−12^).

#### 2.2.3. Venom Allergens

Six venom allergen-like proteins were identified in the *C. fuscescens* proteome, all of which belong to the cysteine-rich secretory protein (CRISP), allergen V5/Tpx-1-related protein family. CRISPs are important components of some snake venoms and act to block smooth muscle contraction or cyclic nucleotide-gated (CNG) ion channels (reviewed in [60]); however these symptoms have not yet been reported for jellyfish venoms. The CRISP protein family is very widespread, including venom allergen 5 (Ves 5) from vespid wasps and venom allergen 3 (Ves 3) from fire ants as well as the plant pathogenesis-related (PR) protein family. All venom allergens identified in the *C. fuscescens* venom proteome were highly homologous to PR-like proteins, five to PRY3-like proteins from *H. vulgaris* and one to a PRY2-like protein from a fungus (Table 2). The PRY2-like protein contained CRISP and CAP domains in combination with a ShKT domain which may indicate a toxic function as discussed in Section 2.3.

#### 2.2.4. Other Potential Toxin Proteins Identified in the *C. fuscescens* Venom Proteome

In addition to proteases, pore-forming toxins and venom allergens, other proteins identified in the *C. fuscescens* proteome included three C-type lectins, one glycoside hydrolase and a protease inhibitor. Examples from all of these protein families have been identified in the venoms of other organisms. C-type lectins have been identified in the venom of snakes, where they act as anticoagulants, procoagulants and agonists/antagonists of platelet activation [61]; beta-hexosaminidases (glycoside hydrolases) have been identified in spider venom where they may act as spreading agents [62]; and Kunitz-type protein inhibitors have been found in the venoms of snakes [63,64], sea anemones [65,66], cone snails [67] and scorpions [68]. The precise role of these toxin families in jellyfish envenomation remains unknown. However, their presence in *C. fuscescens* venom suggests that in addition to developing novel molecular strategies for achieving prey immobilisation/death or defence (e.g., pore-forming toxins), they also employ similar molecular techniques to other venomous organisms. For example, the glycoside hydrolase identified in the *C. fuscescens* venom proteome contained a glycoside hydrolase superfamily feature (IPR017853) but also a chitobiase/beta-hexosaminidase domain (IPR029018) which is involved in the degradation of chitin, the most common component of exoskeletons of arthropods and insects [69]. In the *C. fuscescens* venom this protein could act in the degradation of chitin from crustaceans and other organisms that are commonly the prey of jellyfish [70].

### 2.3. Putative Venom Proteins with ShKT Domains

Two potential toxin proteins, a C-type lectin (comp13880_c0_seq1) and a venom allergen (comp12264_c0_seq1), identified in the *C. fuscescens* venom proteome were also distinguished by the presence of a ShKT domain (IPR003582). ShKT domains are found in a group of potent potassium (K^+^) channels blockers that were originally isolated from sea anemones such as BgK from *Bunodosoma granulifera* and ShK from *Stichodactyla helianthus* (reviewed in [71]). Sequence analysis of the transcriptome (see Section 2.4) also identified a further 15 putative proteins incorporating one to four consecutive ShKT domains (Table S2). These proteins were predominantly proteases (14 proteins) plus an additional venom allergen protein. The ShKT domains identified in these proteins all contain the characteristic six Cys “signature” of the ShKT domain and thus are able to form the three disulphide bonds that stabilise the sea anemone toxins [71]. Proteins containing ShKT domains have been previously reported in anthozoans *A. viridis* [24] and *N. vectensis* [72,73], hydrozoans *H. vulgaris* and *Clytia hemisphaerica* [74], scyphozoans *A. aurita* and *S. meleagris* [23] and cubozoans *Malo kingi* [56], *Carukia barnesi* [56] and *C. fleckeri* [40]. Most of these proteins contain the ShKT domains in combination with other domains such as those of zinc- and astacin-metalloproteinases [24,75]. Although not identified in the nematocyst venom, a ShKT sequence has also been identified in an antimicrobial peptide from the mesoglea of *A. aurita* (e.g., aurelin) [76]. Although the role of these domains is unclear, the combination of the ShKT domain with other functionally diverse protein domains may be indicative of a dual function for some toxin proteins; for example prey immobilisation via ShKT-mediated ion channel blockage and proteinase-mediated digestive functions [75].

### 2.4. Putative Venom Proteins Identified Exclusively in the C. fuscescens Transcriptome

Although transcriptomic sequencing of *C. fuscescens* tentacle tissue was primarily used in this study to generate protein sequences for use in proteomics experiments, it also provided an opportunity to identify potential toxins that were not detected using mass spectrometry. While potential toxins identified in this way lack direct evidence of their presence in the venom*,* comparison with proteins identified in the venom of other jellyfish species can strengthen the likelihood that these proteins are also *C. fuscescens* venom proteins. Accordingly, to identify putative toxins from the tentacle transcriptome, we used a BLAST-based toxin identification pipeline previously developed for the box jellyfish, *C. fleckeri* [26]. Transcripts encoding potential toxins were searched against the UniProt animal toxin and venom database [77] and the complete UniProt database using BLASTx. Transcripts with significant similarity to known toxin proteins from the venom database that did not have a higher-scoring hit to a “non-toxin” protein in the UniProt database were then manually filtered for BLAST hit quality; that is, sufficient coverage of homologous regions and the absence of potential assembly artifacts such as chimeras, and those remaining were designated potential toxins. Using this pipeline, 131 potential protein toxins were identified (Table S2), although proteomic or experimental evidence will be required to validate these potential toxins.

Similar to the venom proteome, the majority of transcripts identified in *C. fuscescens* encoded enzymes. Potential enzymes included serine peptidases and metalloproteinases, comparable to the putative enzyme categories recently identified in the tentacle transcriptome of *C. capillata* [35]. Potential metalloproteinases identified in *C. fuscescens* were mainly disintegrin-like and astacin-like metalloproteinases. Disintegrin-like proteins have been identified in the venoms of *S. meleagris* and *O. sambaquiensis* [20,78] where they could cause severe inflammation by disrupting capillary vessels and tissue [20]. Astacin-like metalloproteinases have been identified in the cnidarian venom proteomes of *N. vectensis* [25], *H. vulgaris* [79], *A. digitifera* [54], *S. meleagris* [23] and *C. fleckeri* [26], where they may act as spreading agents or be involved in the proteolytic processing of other venom proteins [80]. An astacin-like metalloproteinase (PMP1) was also identified in the transcriptome of the hydrozoan *Podocoryne carnea*, but not in the nematocyst contents [75]. Although in this case, *in situ* hybridization experiments revealed high levels of expression in medusa buds and digestive structures, suggesting a functional role in development or food digestion rather than envenomation.

Nine phospholipase-like proteins were identified exclusively in the transcriptome of *C. fuscescens*, eight from the A2 subfamily (PLA2) and a single type B phospholipase (PLB). PLA2 proteins are widely identified in jellyfish tissues [81,82], including nematocysts [20,83,84,85,86,87], and cause inflammation, neurotoxicity, myotoxicity, which could explain localized irritation at the sting site and other cytotoxic effects associated with jellyfish envenomation [82,85,88]. PLA2-like proteins were present in the venom proteomes of *O. sambaquiensis* [20], *H. vulgaris* [24,36] and the coral *A. digitifera* [54]. Although not identified in the *C. fuscescens*, *S. meleagris* and *C. fleckeri* proteomes, their abundance in their transcriptomes, and that of *C. capillata* [35], suggests that they could be venom proteins but present in the nematocyst at very low levels or that the dynamic range of the venom proteome inhibits their identification using mass spectrometry.

Four lysosomal acid lipase (LAL)-like proteins were also identified in the *C. fuscescens* transcriptome as potential toxins. LALs (or LIPAs) have been identified in the tentacle transcriptome of *C. capillata* [35] and *C. fleckeri* [26], as well as the venom glands of snakes [89,90]. Although LALs are proteins associated with intracellular metabolism of lipids by degrading cholesterol esters and triglycerides, their functional role in venoms remains unclear.

Two transcripts encoding potential toxins with high similarity to plancitoxin-1-like proteins from *H. vulgaris* were also identified in the transcriptome of *C. fuscescens* and recently in that of *C. capillata* [35]. These toxins were first identified in the venom of the starfish *Acanthaster planci* and possess DNase II activity which preferentially hydrolyses double-stranded DNA during apoptosis and/or in engulfment-mediated DNA degradation [91] and are potently hepatotoxic and lethal to mice [91,92,93]. The translated *C. fuscescens* plancitoxin-1-like transcripts both contained predicted signal peptides and have retained key elements of the plancitoxin-1 family, including conserved Cys residues involved in disulphide bridge formation and His residues in the DNase II active site, suggesting similar biological mechanisms of action [94].

### 2.5. Comparison of C. fuscescens Transcriptome and Proteome With Other Cnidarians

The availability of cost effective transcriptomic and proteomic profiling technology is providing a much better overview of the constituents of cnidarian venoms (Table 4). A previous comparison of venoms from species representing cnidarian Classes Anthozoa, Hydrozoa and Scyphozoa highlighted the variation in the major constituents of anthozoan and medusozoan venoms (scyphozoan and hydrozoan) [24]. Medusozoan venoms from *A. aurita* and *H. vulgaris* were characterized by the presence of large proteins, predominantly proteases and pore-forming toxins, while the venom of *A. viridis* was abundant in low molecular weight neurotoxins that are potent Na^+^ and K^+^ channels blockers. However, this distinction based on venom composition is somewhat challenged by the increasing identification of higher molecular weight proteins such as enzymes and cytolysins in the venoms of anthozoans [24,54]. Indeed, a wide diversity of serine proteases, metalloproteinases and enzyme inhibitors appear to be major components of all cnidarian venoms as shown in Table 4. Similarly, pore-forming toxins originally found in cubozoan venoms, are increasingly being identified in other medusozoan venoms including the hydrozoan *H. vulgaris* [24], several scyphozoans including *A. aurita* [24], *C. quinquecirrha* [52], *C. capillata* [35] and *C. fuscescens*, and also in anthozoans such as *A. viridis* [24] and *A. digitifera* [54]. The expansion of this toxin family illustrates that toxin families are not necessarily restricted to certain taxonomic groups but have a common presence throughout cnidarian venoms. Therefore, the variable toxicity of cnidarian venoms and the wide range of symptoms they elicit in humans may be associated with different levels of toxin expression rather than the presence or absence of specific toxin families.

In the evolutionary history of cnidarians, different strategies to efficiently capture prey and/or deter predators may have emerged through variation of venom composition, the number of toxin isoforms and the level of expression of these proteins. Comparative analysis of the venom proteomes of jellyfish species is now becoming more feasible and differences in the protein composition of venoms can be a guide for identifying proteins with potential therapeutic applications or those which are responsible for the most severe symptoms of jellyfish stings. The description here of the *C. fuscescens* venom proteome further contributes to the understanding of the cnidarian venom, its potential effects on humans, our ability to treat jellyfish stings and the prospective exploitation of these molecules as sources of novel bioactivities.

## 3. Conclusions

In this project, the tentacle transcriptome and venom proteome of *C. fuscescens* were correlated to identify putative toxins and related venom components. The transcriptome constituted an essential tool for the description of the venom composition because it not only provided a species-specific sequence database for protein identifications using proteomic analysis, but also a catalogue of putative proteins with potential toxic activities at the transcript level. The putative toxins identified in this study show the molecular diversity of jellyfish venoms and reflect the conservation of core toxin families across the cnidarian Classes. These new data can also be used for novel protein/peptide discovery or further comparative studies to increase our understanding of the toxinology of venomous marine animals. Moreover, the identification of diverse proteins with potential toxic roles such as enzymatic and pore-forming mechanisms may also explain, at least partially, their contribution in envenoming processes. In this way, the outcomes of this study can help in improving the current strategies for treatment of human envenomation.

## 4. Materials and Methods

### 4.1. Jellyfish Collection

Live *C. fuscescens* jellyfish were originally collected off the coast of Newport (Newport, OR, USA) and shipped to the Tennessee Aquarium (Chattanooga, TN, USA) where they have reproduced since 2006 [97]. Jellyfish were kept in pseudokreisel aquariums and fed twice daily with live brine shrimps, bloodworms and other invertebrates. In August 2013, 20 mature specimens were selected for this study and fasted for 24 h prior to sampling to avoid contamination from food. All animal handling procedures were approved by the Animal Health and Welfare Committee from the Tennessee Aquarium and the Tennessee Aquarium Conservation Institute (TNACI) under the proposal number 14-02, approved at 10 June 2013.

### 4.2. cDNA Library Construction and Illumina Sequencing

The fishing tentacles of one *C. fuscescens* specimen were manually excised, cut into pieces (5 cm long) and flash-frozen in dry ice. Tentacle samples were shipped to LC Sciences (Houston, TX, USA) for RNA extractions and Next Generation Sequencing. Total RNA was extracted using the RNeasy^®^ Mini Kit (Qiagen, Valencia, CA, USA) as specified by the manufacturer and the integrity was assessed using an Agilent RNA 6000 Nano chip and the Agilent Bioanalyzer 2100 system (Agilent Technologies, Santa Clara, CA, USA). High-quality total RNA was then used to construct a cDNA library using the TruSeq^®^ stranded mRNA Sample Prep Kit (Illumina, San Diego, CA, USA) and sequenced using massively parallel synthesis in one lane on an Illumina HiSeq 2000 system. All sequence data was deposited in the NCBI Short Read Archive under the accession number SRP070629.

### 4.3. De Novo Transcriptome Assembly

After Illumina sequencing, the quality of raw sequence data was assessed using FastQC (version 0.9.2) [98]. Illumina adapter sequences and low quality bases (Phred score > 32) were then removed from the sequence reads using Trimmomatic [99,100]. Reads shorter than 36 base pairs were discarded and the quality of filtered data was re-evaluated using FastQC. After quality control, paired-end sequences were *de novo* assembled into contigs using Trinity (version r20140413p1) [32] using the default parameters. The relative abundance of each transcript was estimated by mapping the raw sequence reads back to the transcriptome assembly using RSEM (version 1.2.12) [101]. Calculated values of Transcripts Per Million (TPM) were obtained for each transcript using the Expectation-Maximization algorithm as a statistical model.

### 4.4. Functional Annotation of Assembled Transcriptome

In order to identify homologous proteins, *C. fuscescens* transcripts were aligned to sequences available in a set of public databases using the tBLASTx and BLASTx algorithms (*E*-value cutoff of 1× 10^−5^) [102]. Searches were conducted against public and custom-made databases including (a) the Swiss-Prot database (as at 1 October, 2013); (b) Cnidarian protein sequences from the GenBank non-redundant (nr) protein database; (c) the complete genomes and transcriptomic data sets of *H. vulgaris* and *N. vectensis* from the Metazome project [103]; and (d) the UniProt animal toxin and venom database [77,104]. The transcriptome was functionally annotated using InterProScan (version 5) [105] and Blast2GO (version 2.5) [34]. Proteins were then classified into defined categories: “Molecular function” (MF), “Biological process” (BP) and “Cellular component” (CC) according to the Gene Ontology (GO) terms defined by the GO project [106]. Signal peptide sequences were detected using SignalP (version 4.1) [44] and transmembrane helices were predicted with TMHMM (version 2.0) [107] or TMPred (version 1.0) [108] using the default search parameters. Sequence analyses were performed using the Geneious software (version R7.1.5) [109]. Multiple sequence alignments were performed using MUSCLE (version 3.8) [110] and visualized using Jalview (version 2.8) [111]. To generate protein databases for proteomic analyses, predicted coding regions (CDS) of transcripts were identified using the ESTScan program (version 3.0) [112] using a cnidarian specific scoring matrix generated previously [26]. For the identification of potential toxins not identified during proteomic analysis, a BLAST-based bioinformatics pipeline was used as previously described [26]. Briefly, transcripts encoding potential toxin proteins were identified using BLASTx (version 2.2.30) against the UniProt animal toxin database [77]. Transcripts with a high-scoring match (bit score > 50) that did not have a better scoring match from the complete UniProt protein database to a non-toxin protein family were designated as a potential toxin. Potential toxins were then manually filtered for hit quality including sufficient coverage of homologous regions and the removal of assembly artifacts such as chimeras.

### 4.5. Venom Sample Preparation for Proteomic Analysis

Fishing tentacles from 20 specimens were excised and placed immediately in 1:10 (*v*:*v*) 35 g·L^−1^ NaCl at 4 °C. Nematocysts were cleaned from tentacle tissue using modified methods previously described [113,114]. For nematocyst isolation, water exchanges were performed every 24 h for 10 days until visible tissue debris was completely discarded. Nematocysts were then cleaned using 100%, 90% and 30% Percoll layers diluted with 35 g·L^−1^ NaCl and centrifugation (300× *g*, 4 °C, 1 h). Cleaned nematocysts were washed thoroughly with 35 g·L^−1^ NaCl and recovered by centrifugation (3000× *g*, 4 °C, 3 min).

Venom was extracted by chemically-induced discharge of nematocysts using dithiothreitol (DTT) (Sigma, St. Louis, MO, USA) in a procedure slightly modified from that previously published [37]. In brief, cleaned nematocysts were washed with 5 mM sodium phosphate buffer, pH 7.5 and recovered by centrifugation (3000× *g*, 4 °C, 3 min). Nematocyst pellets were then resuspended 1:6 (wet *w*:*v*) in SDS-sample buffer [115] containing 200 mM DTT and incubated at room temperature for 30 min. Discharge of nematocyst capsules (>90%) was confirmed microscopically and debris was removed by centrifugation (20000× *g*, 4 °C, 10 min). Soluble venom in supernatant was collected and used for further experiments.

### 4.6. SDS-PAGE and In-Gel Digestion

Two replicates of venom samples (7.5 μL) were heated (95 °C, 5 min) and loaded onto 15% reducing SDS-PAGE gels. Electrophoresis was performed according to Laemmli [115] using a Mini-PROTEAN II system (Bio-Rad, Hercules, CA, USA) at 170 V for 60 min. Proteins were stained using Coomassie Brilliant Blue R-250 (Bio-Rad) and each gel lane was cut into 40 slices using a 1.5 mm × 5 mm GridCutter (Gel Company, San Francisco, CA, USA). Gel fragments were destained twice with 50% acetonitrile in 50 mM triethylammonium bicarbonate buffer (TEAB) (Sigma) for 10 min. The fragments were then dehydrated with 50 mM TEAB followed by 100% acetonitrile and then dried at 37 °C using a vacuum centrifuge. After destaining, cysteine (Cys) residues were reduced by incubation with 10 mM DTT (Bio-Rad) at 60 °C for 30 min. DTT was removed by pipetting and samples were then alkylated with 55 mM iodoacetamide (Bio-Rad) in darkness at room temperature for 30 min. Gel slices were then washed twice with acetonitrile and TEAB and dried at 37 °C. Proteins in the gel fragments were digested by incubation with 0.4 μg trypsin (trypsin from porcine pancreas, Sigma) in 9% acetonitrile and 50 mM TEAB buffer at 37 °C overnight. The digest supernatant was recovered and remaining peptides were extracted from gel slices by washing with 50 mM TEAB, 100% acetonitrile and 5% formic acid. All supernatants containing tryptic peptides were pooled, dried at 45 °C for 8–10 h and stored at −20 °C until further analysis.

### 4.7. Tandem Mass Spectrometry

Dried peptides were resuspended in 20 μL 0.1% formic acid [aq]/2% acetonitrile, centrifuged at 12,000× *g* for 1 min and analysed by LC-MS/MS on a Shimadzu Prominence Nano HPLC (Kyoto, Japan) coupled to a TripleTOF 5600 mass spectrometer (ABSCIEX, Concord, ON, Canada) equipped with a nano electrospray ion source. Two μL of the peptide mix was injected onto a 50 mm × 300 μm C18 trap column (Agilent) at 20 μL/min. The samples were de-salted on the trap column for 5 min using 0.1% formic acid [aq] at 20 μL/min. The trap column was then placed in-line with an analytical nano-HPLC column (150 mm × 75 μm C18, 5 μm; Vydac, Hesperia, CA, USA) for mass spectrometry analysis. A linear gradient of 1%–80% solvent B (90/10 acetonitrile/0.1% formic acid [aq]) over 120 min at an 800 nL/minute flow rate, followed by a steeper gradient from 40% to 80% solvent B in 5 min, was used for peptide elution. The ionspray voltage was set to 2000 V, declustering potential 100 V, curtain gas flow 25, nebuliser gas 1 (GS1) 10 and interface heater at 150 °C. 500 ms full scan TOF-MS data was acquired followed by 20 × 50 ms full scan product ion data in an Information Dependant Acquisition (IDA) mode. Full scan TOF-MS data were acquired over the mass range 350–1800 and for product ions 100–1800. Ions observed in the TOF-MS scan exceeding a threshold of 100 counts and a charge state of +2 to +4 were set to trigger the acquisition of product ion spectra for a maximum of 20 of the most intense ions. The data was acquired and processed using Analyst TF 1.5.1 software (ABSCIEX, Concord, ON, Canada). All proteomics data was deposited in the MassIVE repository (Center for Computational Mass Spectrometry, University of California, San Diego, CA, USA) under accession number MSV000079527.

### 4.8. Spectral Searches and Bioinformatics Analysis

Searches were performed using ProteinPilot (version 4, ABSCIEX) using the following parameters: allowing for methionine oxidation as a variable modification, carbamidomethylation as a fixed modification, two missed cleavages, charge states +2, +3 and +4 and trypsin as the enzyme. Searches were conducted against the translated protein sequences from *C. fuscescens* transcripts described above. Spectral data was also searched against a database composed of cnidarian protein sequences from GenBank (122,112 sequences as at 1st November, 2015) and unique protein identifications determined by sequence comparison to proteins identified during spectral searches of the *C. fuscescens* predicted protein dataset. Proteins were grouped using ProteinPilot’s ProGroup algorithm, single peptide identifications were not considered and only proteins containing at least one unique, significant peptide identification were reported. Searches were also conducted with X! TANDEM Jackhammer TPP (version 2013.06.15.1) [116] using the same database and the following parameters: enzyme = trypsin; precursor ion mass tolerance = ±0.1 Da; fragment ion tolerance = ±0.1 Da; fixed modifications = carbamidomethylation; variable modifications = methionine oxidation; number of missed cleavages allowed = 2; allowed charge states = +2 and +4; and “k-score” as the scoring algorithm.

## Figures and Tables

**Table 1:** Description of *de novo* assembly and analysis of the *C. fuscescens* tentacle transcriptome.

Assembly	Count
Raw reads (paired-end)	26,991,925
After cleaning	17,319,746
Contigs	30,317
Average length ± SD	628.70 ± 840.07
Length (min and max)	201 to 31,945
GC content	40.42%
Raw reads mapped to contigs	97.69%
**CDS**	**Count**
Containing a coding region	23,534 (78%)
Transcripts with significant BLAST hit (1 × 10^−5^)	16,925 (72%)
With homologues in databases:	
GenBank non-redundant Cnidarian protein sequences	15,987 (53%)
*H. vulgaris*	14,261 (47%)
SwissProt	13,375 (44%)
*N. vectensis*	12,144 (40%)
Uniprot animal toxin and venom	549 (2%)
**Sequence analysis**	**Count**
Returning GO term	11,586 (49%)
GO terms returned:	
Molecular function	8265 (35%)
Biological process	4768 (20%)
Cellular component	2173 (9%)
Predicted proteins with signal sequences *	1012 (4%)
Predicted proteins with two or more transmembrane helices	641 (2%)

* SignalP on top hit from SwissProt returned 1666 (7%).

**Table 2:** Putative toxins and venom-related proteins identified in the *C. fuscescens* venom proteome using MS/MS.

Transcript	Category	Unique Peptides (*n*)	Coverage (%)	InterProScan Protein Feature	Signal Peptide (Yes/No)	Transmembrane Domain (Yes/No)	BLAST Analysis	
							Protein Identity	Species of Closest Homology
comp13691_c0_seq2	Metalloprotease	18	76.6	Peptidase M1, alanine aminopeptidase	n	n	Aminopeptidase N-like	*H. vulgaris*
comp12218_c0_seq1	Metalloprotease	16	76.2	Peptidase M13	n	y (27–49)	Endothelin-converting enzyme 1-like	*H. vulgaris*
comp13767_c0_seq1	Metalloprotease	10	80.4	Peptidase M13	y (1–29)	y (12–29)	Endothelin-converting enzyme 2-like	*H. vulgaris*
comp11996_c0_seq1	Metalloprotease	8	62.2	Peptidase M1, alanine aminopeptidase	n	n	Endoplasmic reticulum aminopeptidase 1-like	*H. vulgaris*
comp11571_c0_seq2	Metalloprotease	8	75.4	Peptidase M13	n	n	Endothelin-converting enzyme 1-like	*H. vulgaris*
comp10942_c1_seq1	Metalloprotease	7	71.0	Peptidase M13	n	y (31–53)	Endothelin-converting enzyme 1-like	*H. vulgaris*
comp12208_c0_seq1	Metalloprotease	6	61.0	Peptidase M14, carboxypeptidase A	y (1–20)	y (292–309)	Carboxypeptidase D-like	*H. vulgaris*
comp9530_c0_seq2	Metalloprotease	6	87.3	Peptidase M13	n	n	Endothelin-converting enzyme 1-like	*N. vectensis*
comp14393_c0_seq1	Metalloprotease	5	68.2	Peptidase M2, peptidyl-dipeptidase A	n	n	Angiotensin-converting enzyme-like isoform	*H. vulgaris*
comp14137_c0_seq1	Metalloprotease	2	42.1	Peptidase M2, peptidyl-dipeptidase A	n	n	Angiotensin-converting enzyme	*H. vulgaris*
comp14070_c0_seq1	Metalloprotease	3	67.3	Peptidase M18	n	y (493–510)	Aspartyl aminopeptidase-like	*Lepisosteus oculatus*
comp13494_c0_seq1	Aspartyl protease	2	39.6	Aspartic peptidase	y (1–17)	y (161–193)	Cathepsin D	*Pteria penguin*
comp12883_c0_seq1	Aspartyl protease	2	48.9	Aspartic peptidase	y (1–18)	y (2–24)	Aspartyl protease	*Placozoa sp.* H4
comp13655_c0_seq2	Serine protease	2	67.8	Peptidase S8/S53	n	y (156–178, 323–339, 748–771)	PC3-like endoprotease variant B isoform X1	*H. vulgaris*
comp13207_c0_seq1	Pore-forming toxin	6	75.4	Delta-endotoxin, *N*-terminal	y (1–19)	y (104–125)	Toxin TX2	*A. aurita*
comp12925_c0_seq1	Pore-forming toxin	8	76.0	Delta endotoxin, *N*-terminal	n	y (15–37)	Uncharacterized protein LOC105843890	*H. vulgaris*
comp13855_c0_seq5	Venom allergen	37	83.0	Cysteine-rich secretory protein, allergen V5/Tpx-1-related	n	n	Cell wall protein PRY3-like	*H. vulgaris*
comp13672_c0_seq1	Venom allergen	18	81.4	Cysteine-rich secretory protein, allergen V5/Tpx-1-related	n	n	Cell wall protein PRY3-like	*H. vulgaris*
comp13791_c0_seq1	Venom allergen	15	75.2	Cysteine-rich secretory protein, allergen V5/Tpx-1-related	n	n	Cell wall protein PRY3-like	*H. vulgaris*
comp13791_c0_seq3	Venom allergen	9	70.6	Cysteine-rich secretory protein, allergen V5/Tpx-1-related	n	n	Cell wall protein PRY3-like	*H. vulgaris*
comp13342_c1_seq2	Venom allergen	2	76.1	Cysteine-rich secretory protein, allergen V5/Tpx-1-related	n	n	Cell wall protein PRY3-like	*H. vulgaris*
comp12264_c0_seq1	Venom allergen	2	81.7	Cysteine-rich secretory protein, allergen V5/Tpx-1-related	y (1–20)	y (7–29)	PRY2-like protein	*Pyronema omphalodes* CBS 100304
comp13629_c0_seq1	C-type lectin	194	85.0	C-type lectin	y (1–20)	y (344–362)	Golgi-associated plant pathogenesis-related protein 1	*H. vulgaris*
comp13792_c0_seq2	C-type lectin	44	70.0	C-type lectin	n	y (96–116)	Golgi-associated plant pathogenesis-related protein 1	*H. vulgaris*
comp13880_c0_seq1	C-type lectin	8	56.5	C-type lectin	y (1–21)	y (348–368)	Golgi-associated plant pathogenesis-related protein 1	*H. vulgaris*
comp13219_c0_seq1	Glycoside hydrolase	18	59.4	Beta-hexosaminidase	y (1–20)	y (416–438)	beta-hexosaminidase subunit alpha-like isoform X1	*H. vulgaris*
comp7130_c0_seq1	Enzyme inhibitor	4	64.0	Peptidase S8/S53 domain	y (1–19)	y (484–507)	Tripeptidyl-peptidase 1-like	*H. vulgaris*

**Table 3:** BLAST homology of CfusTX-1 from *C. fuscescens* and pore-forming toxins from other jellyfish.

Toxin ID	Jellyfish Species	BLAST		UniProt Accession No.	Reference
		Identity (%)	*E*-Value		
TX2 *	*A. aurita*	48	2 × 10^−124^	I3VAS2	UniProt
TX1 *	*A. aurita*	37	2 × 10^−96^	I3VAS1	UniProt
CfTX-A	*C. fleckeri*	25	1 × 10^−27^	T1PRE3	[45]
CrTX-A	*Carybdea rastoni* ^1^	25	3 × 10^−26^	Q9GV72	[48]
CfTX-1	*C. fleckeri*	24	9 × 10^−26^	A7L035	[46]
CfTX-2	*C. fleckeri*	24	3 × 10^−27^	A7L036	[46]
CqTX-A	*Chiropsalmus quadrigatus* ^2^	24	9 × 10^−25^	P58762	[50]
CaTX-A	*Carybdea alata* ^3^	24	1 × 10^−23^	Q9GNN8	[49]
CfTX-Bt	*C. fleckeri*	24	2 × 10^−16^	W0K4S7	[45]
CfTX-B	*C. fleckeri*	23	1 × 10^−26^	T1PQV6	[45]
MkTX-A1 *	*M. kingi*	20	6 × 10^−10^	D2DRC0	[56]
MkTX-A2 *	*M. kingi*	20	1 × 10^−9^	D2DRC1	[56]

* Predicted proteins; ^1^ renamed as *Carybdea brevipedalia* by Toshino *et al.* [57]; ^2^ renamed as *Chironex yamaguchii* by Lewis and Bentlage [58]; ^3^ renamed as *Alatina moseri* by Gershwin [59].

**Table 4:** Diversity of major potential toxin families identified in cnidarian venoms using transcriptomic and/or proteomic (MS/MS) analyses.

Toxin Family	Class Scyphozoa				Class Cubozoa			Class Hydrozoa		Class Anthozoa			
	*Cyanea capillata*	*Stomolophus meleagris*	*Aurelia aurita*	*Chrysaora fuscescens*	*Chironex fleckeri*	*Malo kingi*	*Carukia barnesi*	*Olindias sambaquiensis*	*Hydra vulgaris*	*Anemonia viridis*	*Nematostella vectensis*	*Acropora digitifera*
Proteinases	(E) Cysteine protease; (T) astacin, zinc and matrix metallo-proteinases, serine proteases; ECE-1	(T) Zinc metallo-proteinases	(T) Zinc metallo-proteinases; ECE-1	(T, P) Serine peptidases, zinc metallo-proteinases, ECE-1 and -2	(T,P) Serine peptidases; astacin and zinc metallo-proteinases; (P) ECE-2 and 2-like	(E) Serine peptidases; carboxy-peptidases; metalloproteases	(E) Serine peptidases	(P) Serine peptidases; zinc metallo-proteinases	(P) Serine peptidases; zinc metallo-proteinase; (T, P) ECE-1	(T) ECE-1	(T) Astacin-like metallo-proteinase	(T, P) Serine peptidases; astacin and other metallo-proteinases
Lipases	(E, T) PLA2; (T) PLD; LALs	(T) PLA2 and PLB1	(T, P) PLA2	(T) LALs; PLA2 and PLB2	(T) LALs; PLA2	-	-	(P) PLA2	(E, T, P) PLA2	-	(T)	(T, P) LALs; endothelial lipase; PLB; PLA1; PLA2
Deoxyribonu-cleases	(T) Plancitoxin-like	-	-	(T) Plancitoxin-like	-	-	-	-	(E) Plancitoxin-like	-	-	-
Cytolysins	(E) Hemolysin C	(T) Hemolysins (homologues to ryncolin, veficolin, hemolysin hlyIII)	-	-	-	-	-	(P) AvTX-60A and PsTX-like	(E) Actinoporins and hydralysins	(T) Actinoporin-like	-	(T, P) Bandaporin; actinoporin; urticinatoxin
Pore-forming (cnidarian toxin family)	(T) CfTX-like; (P) CcTX-1	-	(T,P) ^1^ AaTX-1 and -2; CaTX-like	(T, P) CfusTX-1	(T, P) CfTXs	(E) MkTXs (CfTX-like)	-	-	(T, P) CaTX-like	(T) CaTX-like	-	(T, P) CfTX-1-like
Pore-forming (MAC-PF)	-	-	(T, P)	-	-	-	-	-	(T, P)	(T, P)	-	-
CRISPs	-	-	*	(T, P) allergen V5/Tpx-1-related	(T) allergen V5/Tpx-1-related	-	-	-	(E) venom allergen 5	*	(T) venom allergen 5 /PR-1-like	-
C-type lectins	-	(T)	(T)	(T, P)	(T, P)	-	-	-	(T, P)	-	(T)	(T, P)
Protease inhibitors	(T) Kazal-type; Kunitz-type	(T) Kunitz-type	(T) Kunitz-type	(T, P) Kunitz-type	(T, P) Cysteine protease inhibitors; Kunitz-type; Kazal-type	(E) Kazal-type	-	-	(T, P) Cysteine protease inhibitors; Kunitz-type	(P) Kunitz-type	-	(T, P) Kunitz-type
Proteins with ShKT domains	-	(T) 1–4 domains + other protein domains	(T) 1–3 domains + other protein domains	(T) 1–4 domains + other protein domains; (P) CRISP-like protein and C-type lectin	(T, P) + astacin domains	(E)	(E)	-	(E, T, P) + metalloprotease-like protein domains	(T, P)	(T)	-
Neurotoxins (modulators of nicotinic receptors or presynaptic nerve endings)	-	(T) Botulinum neurotoxins; α-latrocrusto-toxin-Lt1a	-	-	-	-	-	(P) κ-4-Bungarotoxin; α-latrocrusto-toxin-Lt1a	-	-	-	-
Neurotoxins (K^+^ channel blockers)	-	-	-	-	-	-	-	-	(E) Kalicludine-like	(T, P) ShK toxins, BDS-like	-	-
Neurotoxins (Na^+^ channel blockers)	-	-	-	-	-	-		-	-	(T, P) Av1, Av2 and Av3	(T) Nv1	(T, P) Av1-like
Associated References	[35,43,53]	[23]	[24]	Present work	[26,37,40]	[56]	[56]	[20]	[24,36,79]	[5,24]	[25,72,73,95,96]	[54]

(E) EST library, (T) transcriptomic or (P) venom proteome analysis; ^1^ GenBank Accession No. AFK76348 and AFK76349; * Present but excluded as potential toxins.

## Supplementary Materials

The following are available online at www.mdpi.com/2072-6651/8/4/102/s1. Figure S1: Coverage and length distribution of transcripts from *C. fuscescens* tentacle transcriptome. (A) Coverage of assembled transcripts after mapping of raw sequences back to the assembly using RSEM; (B) Length distribution of transcript. Table S1: Protein identifications from tandem mass spectrometry analysis of *C. fuscescens* venom, Table S2: *C. fuscescens* tentacle transcripts with high-scoring BLASTx matches in the UniProt animal venom and toxin database.
