# Supplementary material for: Tentacle Transcriptome and Venom Proteome of the Pacific Sea Nettle, Chrysaora fuscescens (Cnidaria: Scyphozoa)
Source: Toxins (Basel). 2016 Apr 5;8(4):102. doi: 10.3390/toxins8040102 (PMC4848628; doi:10.3390/toxins8040102)
Supplement: Supplementary file 1 [file toxins-08-00102-s001.zip › toxins-116439-supplementary.pdf]

Supplementary Materials: Tentacle Transcriptome and Venom Proteome of the Pacific Sea Nettle, *Chrysaora fuscescens* (Cnidaria: Scyphozoa)

Dalia Ponce [1,†], Diane L. Brinkman [2,*,†], Jeremy Potriquet [3] and Jason Mulvenna [3,4,*,†]

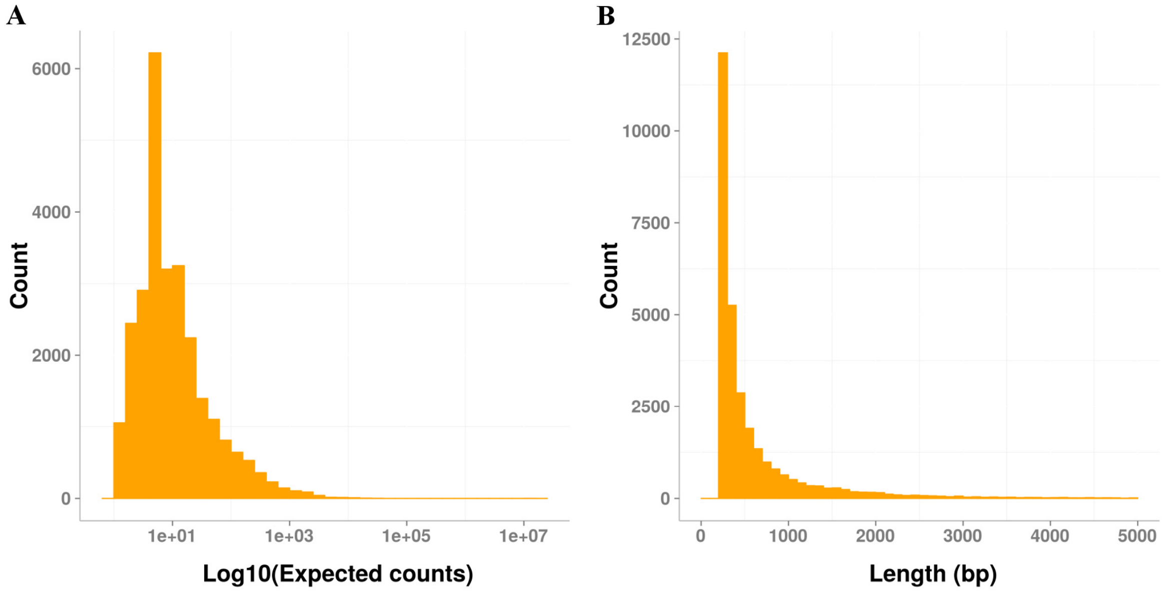

Figure S1. Coverage and length distribution of transcripts from *C. fuscescens* tentacle transcriptome. (**A**) Coverage of assembled transcripts after mapping of raw sequences back to the assembly using RSEM; (**B**) Length distribution of transcript.
